# Oesophageal Conduit – a Cause of Diffuse Mediastinal Uptake on Thyroid Scintigraphy

**DOI:** 10.4274/Mirt.147

**Published:** 2013-12-10

**Authors:** Luke I. Sonoda, Kottekkattu K. Balan

**Affiliations:** 1 Mount Vernon Hospital, Paul Strickland Scanner Centre, Middlesex, United Kingdom; 2 Addenbrooke’s Hospital, Nuclear Medicine, Cambridge, United Kingdom

**Keywords:** Esophagectomy, scintigraphy, mediastinum, 99mTc-pertechnetate, tissue distribution

## Abstract

99mTc-pertechnetate scintigraphy plays an essential role in the management of a variety of thyroid and parathyroid disorders. The authors report an unusual case of mediastinal tracer distribution of 99mTc-pertechnetate and 99mTc-MIBI in relation to an oesophageal conduit following oesophago-gastrectomy and reconstructive surgery on thyroid scintigraphy. This is a rare but important cause of diffuse mediastinal uptake on thyroid scintigraphy. An awareness of abnormal anatomy as well as altered physiological tracer uptake would help to avoid any diagnostic pitfall.

**Conflict of interest:**None declared.

## INTRODUCTION

In the management of thyroid and parathyroid disorders, 99mTc-pertechnetate scintigraphy and 99mTc-MIBI scintigraphy are commonly used to image and localise the lesions. However, the uptake of these tracers may not be organ specific to the target tissues of interest. It is therefore important to recognize altered physiological and pathological tracer uptake patterns in the event of abnormal anatomy, often due to previous surgery. We present a patient with an altered tracer uptake due to previous oesopho-gastric surgery in 99mTc-pertechnetate and 99mTc-MIBI scintigraphy.

## CASE REPORT

**Figure 1:**
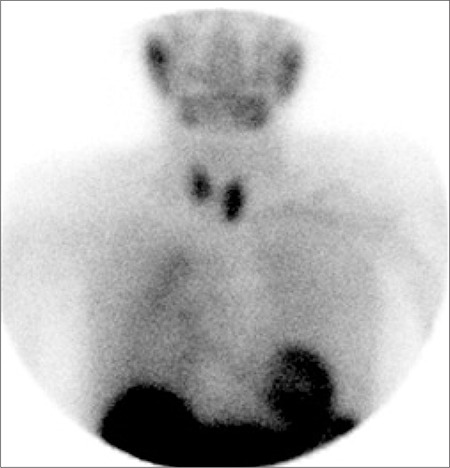
99mTc-MIBI scan showing uniform tracer uptake in the thyroidwith a focus of abnormal activity below the lower pole of the left lobe. Inaddition there is faint, diffuse increased uptake in the mediastinum to theright of midline.

**Figure 2:**
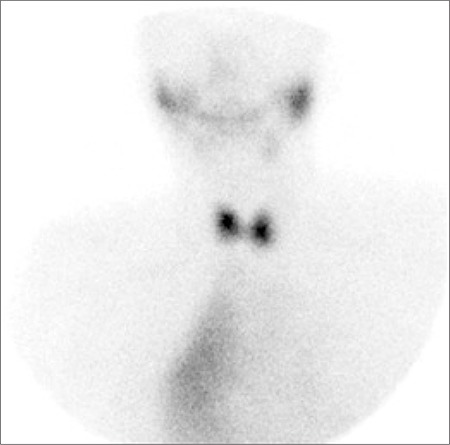
99mTc-pertechnetate scan showing no abnormal uptake in thelower pole of the left lobe of thyroid. Together with the appearance in Fig.1, the finding confirms the presence of a parathyroid adenoma. There isalso persistent mediastinal activity suggesting uptake in a holow organ.

**Figure 3:**
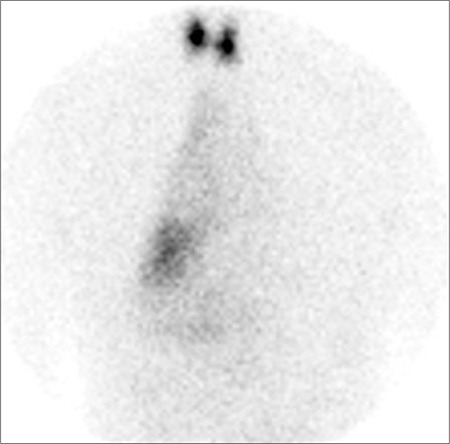
Repeated 99mTc-pertechnetate study following a glass of waterto drink. Albeit with reduced intensity, the mediastinal uptake remains unchanged.

**Figure 4:**
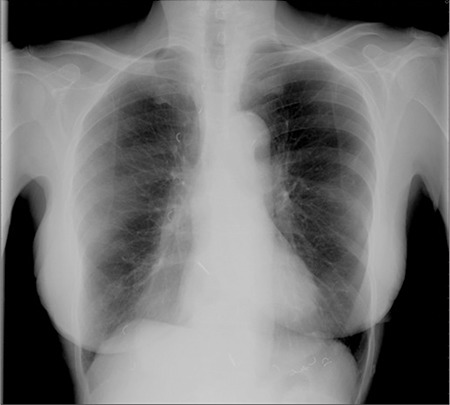
Frontal chest radiograph showing oesophageal conduit withmultiple surgical clips in the mediastinum to the right of midline.

A 76-year-old woman with elevated serum calcium (2.64 mmol/l) and PTH (177 pmol/l) was referred to the nuclear medicine department for parathyroid imaging in order to pre-operatively localise a parathyroid adenoma. She underwent early and delayed scanning of the neck and mediastinum after intravenous injection of 600 MBq 99mTc-methoxyisobutylisonitrile (MIBI). The images showed uniform tracer uptake in the thyroid with a focus of abnormal activity below the lower pole of the left lobe. In addition, faint, diffusely increased uptake was noted in the mediastinum to the right of midline (Figure 1). In an effort to exclude multinodular goitre as a cause of false positive parathyroid adenoma, a 99mTc-pertechnetate study was performed on a separate day. This showed no abnormal uptake in the lower pole of the left lobe of thyroid thereby confirming the presence of a parathyroid adenoma. Further, there was persistent mediastinal activity, suggesting uptake in a hollow organ (Figure 2). This uptake remained unchanged, albeit with reduced intensity, on repeat imaging after the patient was given a glass of water to drink (Figure 3). Review of the chest radiograph (Figure 4) and case notes revealed that the patient had previously undergone oesophago-gastrectomy and reconstructive surgery for a gastric pathology. It was therefore concluded that the mediastinal uptake of pertechnetate observed in this patient was presumably due to ingested salivary activity in the oesophageal conduit. As to the mediastinal 99mTc-MIBI uptake, this has been earlier reported in a patient with oesophagitis (1).

## LITERATURE REVIEW AND DISCUSSION

^99m^Tc-pertechnetate scintigraphy has been invaluable in the management of a variety of thyroid and parathyroid disorders (2). A good understanding of the physiological distribution of the tracer is however important in identifying artefacts since most of them are caused by normal secretion into body fluids or tissues. An awareness of the pathological conditions that produce unusual appearances may also help to avoid diagnostic pitfalls. 

A wide spectrum of potentially misleading physiological and non-physiological process may cause mediastinal uptake on 99mTc-pertechnetate scintigraphy, most common conditions include substernal extension of the thyroid, secreted activity in the oesophagus, intrathoracic thyroid tissue, metaplastic gastric mucosa (Barrett’s oesophagus) and gastric mucosa in hiatus hernia (3,4,5,6). Other uncommon causes are: metastatic thyroid carcinoma, ingested activity in the oesophagus in achalasia, tumours with functioning thyroid tissue, thymoma and intrathoracic gastric cyst (7,8,9,10,11). 

^99m^Tc-MIBI is commonly used in myocardial perfusion scintigraphy (12) and in parathyroid scintigraphy (13). More recently it has also been applied in breast scintigraphy (14). It is considered that MIBI is sequestered within the mitochondria (15), therefore a tissue with a large number of mitochondria such as myocardial cells and oxyphil cells of abnormal parathyroid glands in primary hyperparathyroidism (16) may take up MIBI avidly and retain longer. Interestingly, it has been long known that patients with oesophagitis may show a ^99m^Tc MIBI uptake along the oesophagus as an incidental finding in cardiac ^99m^Tc MIBI scintigraphy (17). Evaluation of the usefulness of Tc MIBI scintigraphy to detect oesophagitis has been performed with relatively high sensitivity (100%) and specificity (77%) (1). In our case report, the patient had a previous oesophago-gastrectomy and hence was expected to have a degree of reflux oesophagitis.
